# Complications of central venous port systems: a pictorial review

**DOI:** 10.1186/s13244-019-0770-2

**Published:** 2019-08-28

**Authors:** Sibylle Machat, Edith Eisenhuber, Georg Pfarl, Josef Stübler, Claus Koelblinger, Johannes Zacherl, Wolfgang Schima

**Affiliations:** 1 Department of Diagnostic and Interventional Radiology, Sankt Josef Krankenhaus, Goettlicher Heiland Krankenhaus, and Barmherzige Schwestern Krankenhaus, Auhofstrasse 189, 1130 Vienna, Austria; 2 Department of Radiology, Barmherzige Schwestern Krankenhaus, Ried, Austria; 3 Department of Surgery, Sankt Josef Krankenhaus, Vienna, Austria

**Keywords:** Port, Complication, Perforation, Malposition, Thrombosis

## Abstract

Central venous port devices are indicated for patients, who need long-term intravenous therapy. Oncologic patients may require intermittent administration of chemotherapy, parenteral nutrition, infusions, or blood transfusions. A venous port system is composed of a port chamber attached to a central catheter, which is implanted into the central venous system. The subcutaneous location of the catheter chamber improves the patients’ quality of life and the infection rate is lower than in non-totally implantable central venous devices. However, proper implantation, use, and care of a port system are important to prevent short- and long-term complications. Most common early complications (< 30 days) include venous malpositioning of catheter and perforation with arterial injury, pneumothorax, hemothorax, thoracic duct injury, or even cardiac tamponade. Delayed complications include infection, catheter thrombosis, vessel thrombosis and stenosis, catheter fracture with extravasation, or fracture with migration or embolization of catheter material. Radiologic imaging has become highly relevant in intra-procedural assessment and postoperative follow-up, for detection of possible complications and to plan intervention, e.g., in case of catheter migration. This pictorial review presents the normal imaging appearance of central venous port systems and demonstrates imaging features of short- and long-term complications.

## Key points

- Central venous port devices were introduced first in 1982 and are increasingly used especially in oncologic patients
- The ideal position of the catheter tip is the distal superior vena cava
- Complications of port systems are divided into early (≤ 30 days after implantation) and delayed (> 30 days) complications and occur in up to 33%
- Most common complications are infection and catheter-related thrombosis
- Due to possible major complications and low cost of chest radiographs, routine postoperative chest radiography is recommended.

## Introduction

Totally implanted central venous port systems are widely used for chronically ill patients, who need long-term access to central veins for prolonged therapy. In 1982, Niederhuber et al. introduced the present used type of port systems into clinical use, which are usually implanted subcutaneously in the chest wall. The port system is built of a central catheter, which is inserted into a cannulated vein beneath the skin and attached to a port chamber that is placed into a subcutaneous pocket. Access of this totally implanted reservoir is possible with a special needle that allows puncture of the skin and silicone membrane of the port chamber. Chamber puncture has to take place under sterile conditions. Furthermore, patients need no external dressing of the port area and are allowed to pursue normal activities like showering and swimming after needle removal. Due to the totally subcutaneous position, the port devices are invisible and patients are not stigmatized [1–3].

Because of their low rates of extravasation and infection, common indications for permanent venous port systems are administration of vascular noxious medications like chemotherapy and parenteral nutrition [4]. Implantation of central venous port systems is performed in an interventional suite or operating room using fluoroscopic guidance under local anesthesia. After creating a venous access and placing a guide wire, a local anesthetic is administered into the skin and subcutaneous tissue and a pocket for the port chamber is created. Then the catheter is tunneled from the pocket to the guide wire. After dilatation of the tract, the catheter is placed into the punctured vein. The excess part of the catheter is cut and attached to the port device, which is secured with sutures. Skin and subcutaneous tissue above port chamber are also sutured. After implantation, a chest radiograph should be obtained to confirm correct positioning of the venous device or to identify possible immediate complications, respectively [5–8]. Of course, even after uneventful implantation, proper catheter maintenance is necessary to avoid complications, which are reported in up to 27% [9–12]. Overall, contraindications are rare. It has been shown that even in patients with thrombocytopenia, a port implantation is possible [13]. This pictorial review gives an illustrated overview of complications, which may be encountered during and after implantation of central venous port systems (see Table 1), since the knowledge about possible complications represents a prerequisite to avoid them.

**Table 1:** Complications after port implantation

Early complications	
• Malposition: intravenous, cardiac	
• Arrhythmia	
• Perforation and bleeding: hemothorax, mediastinal, cardiac tamponade	
• Arterial malpositioning	
• Pneumothorax	
• Thoracic duct injury	
• Air embolism	
Delayed	
• Infection	
• Venous thrombosis, pulmonary embolism	
• Venous stenosis	
• Catheter pinch-off, fracture and migration	
• Catheter embolization	
• Air embolism	

### Normal imaging appearance after implantation

No universally accepted definition of the ideal position of the catheter tip exists. However, it has been advocated that the catheter tip is ideally located in the distal superior vena cava (SVC) in port systems implanted in the internal jugular or the subclavian vein: the large volume of blood in a wide caliber vein immediately dilutes administered medication and reduces risk of vascular damage. This is especially important in chemotherapeutic drugs, which are administered in solutions with high osmolality. They are known to damage the vascular wall with subsequent possible complications like infection and thrombotic occlusion and narrowing of the venous caliber—hence suboptimal tip position may lead to delayed complications [14].

**Fig. 1:**
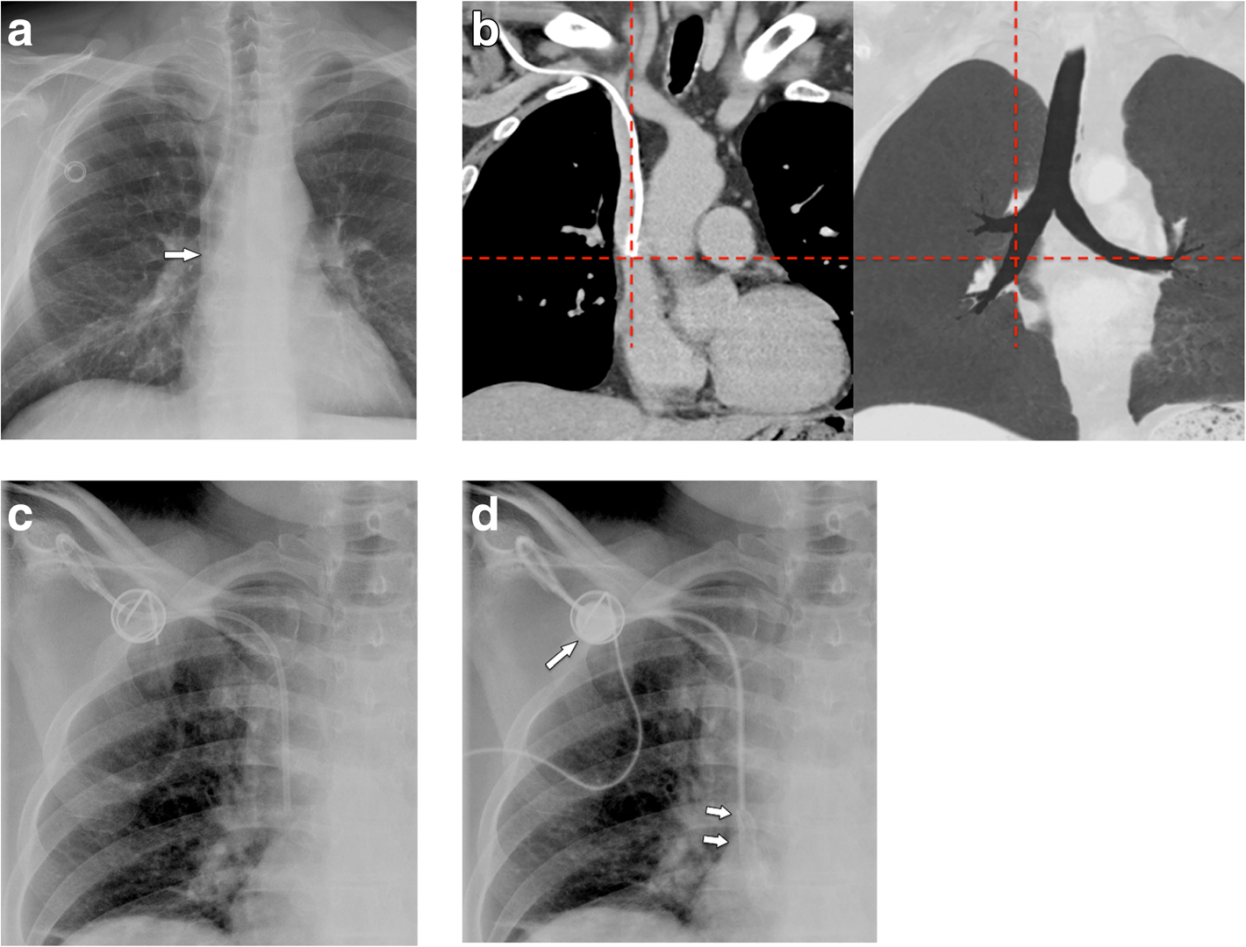
Normal position of implanted port system. **a** Chest radiograph shows the tip of the venous catheter projecting onto the intersection with the intermediate bronchus, which suggests position in the SVC. **b** Coronal MDCT reformation (soft tissue window and minimum intensity projection of lung window) shows that intersection of the tip with intermediate bronchus indicates correct position in the SVC. **c**, **d** Flow confirmation study (**c**) before and (**d**) after injection of contrast. The port chamber is completely opacified (arrow), the venous tube does not show leakage, and contrast material exits freely at the tip flowing in antegrade direction (small arrows)

On chest radiographs, the distal SVC projects over the right main/intermediate bronchus. Thus, placement of the tip at the crossing of the SVC and right main bronchus will provide adequate positioning (Fig. 1a, b). During flow confirmation studies, complete filling of the port chamber with contrast material should be seen. Contrast material fills the venous tube without leakage, coming out of the tip to flow freely in the SVC (Fig. 1c, d).

### Complications

Complications of venous port systems are divided into periprocedural early (≤ 30 days after implantation) and delayed (> 30 days) complications. Complications can be defined as “minor” or “major.” Minor complications are events, which do not require additional surgical or interventional therapy or medical therapy > 24 h, whereas major complications require surgery/intervention, prolonged medical therapy, a hospital stay > 24 h, or even result in death. Hemothorax and pneumothorax are the most likely major complications, based on the severity.

The overall complication rate has been reported to be 7.2–12.5%, with port system infection being most common [2, 15]. With an incidence of 5–18%, catheter-related thrombosis is also relatively common and does not necessarily require catheter explantation. Depending on the need for central access, functional status of catheter system, review of contraindications against anticoagulation, and patient’s condition the further management should be individually discussed [16].

### Early complications

#### Common malpositioning

**Fig. 2:**
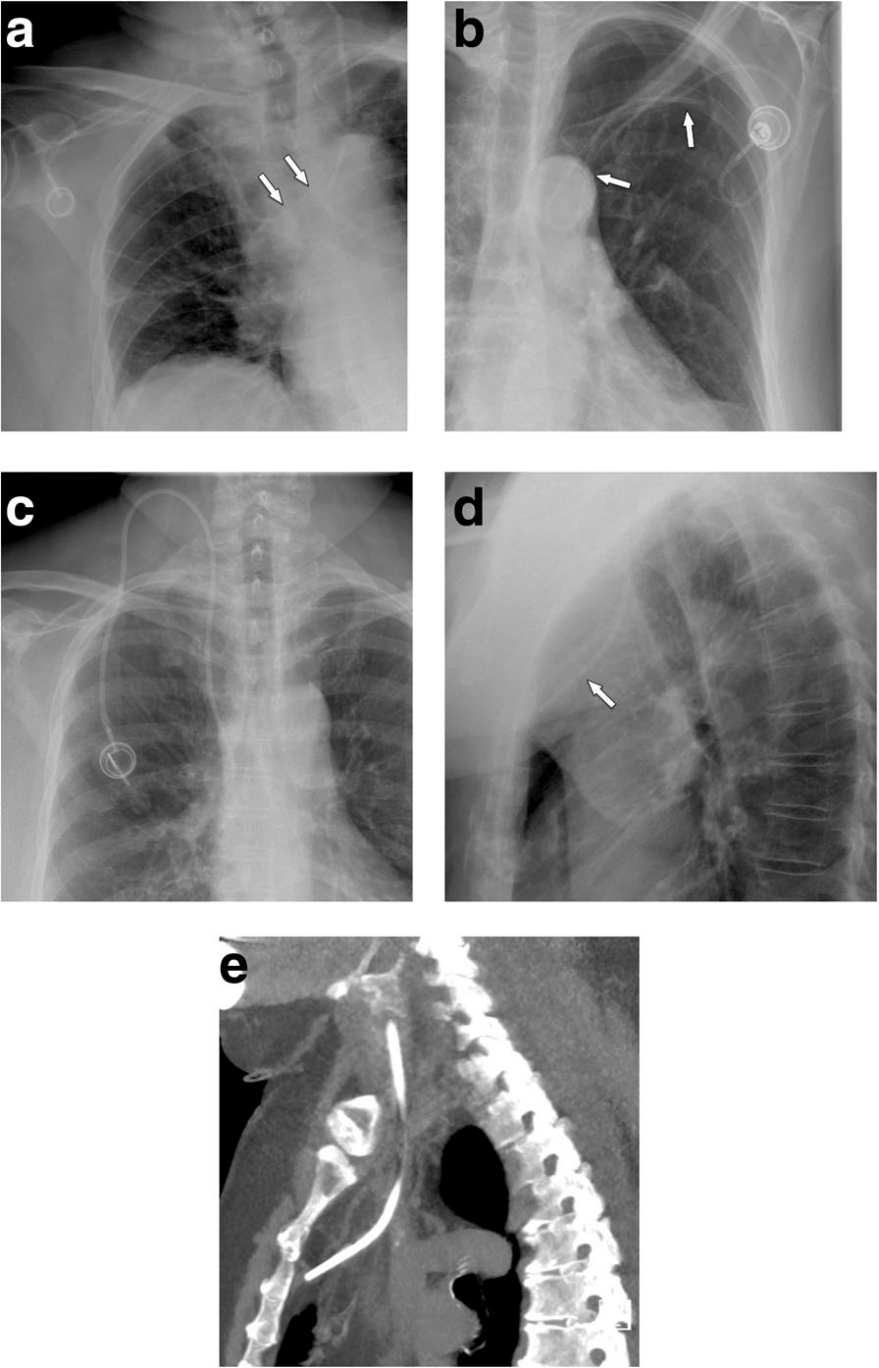
Common venous malpositioning. **a** The venous catheter, implanted via the subclavian vein, enters the contralateral brachiocephalic vein (arrow). **b** Loop formation in the subclavian vein. **c**–**e** Malpositioning in the mammarian vein due to occlusion of the SVC. **c** PA chest film shows normal course of the catheter, which had been implanted into the internal jugular vein. **d** However, the lateral projection shows an abnormal anterior course. **e** Sagittal MIP CT reconstruction confirms malpositioning into the internal mammary vein

Malpositioning of the catheter from the subclavian vein into the ipsilateral internal jugular vein and vice versa does occur, as well as malpositioning into the azygos vein, internal mammary vein, and left superior intercostal vein, etc. (Fig. 2a, b). Such a malposition should be readily recognized during fluoroscopy or on postoperative AP chest radiographs. Only placement into the right internal mammary vein can be difficult to detect on single-plane chest radiographs (Fig. 2c–e).

#### Positioning in the persistent left superior vena cava: a malposition?

**Fig. 3:**
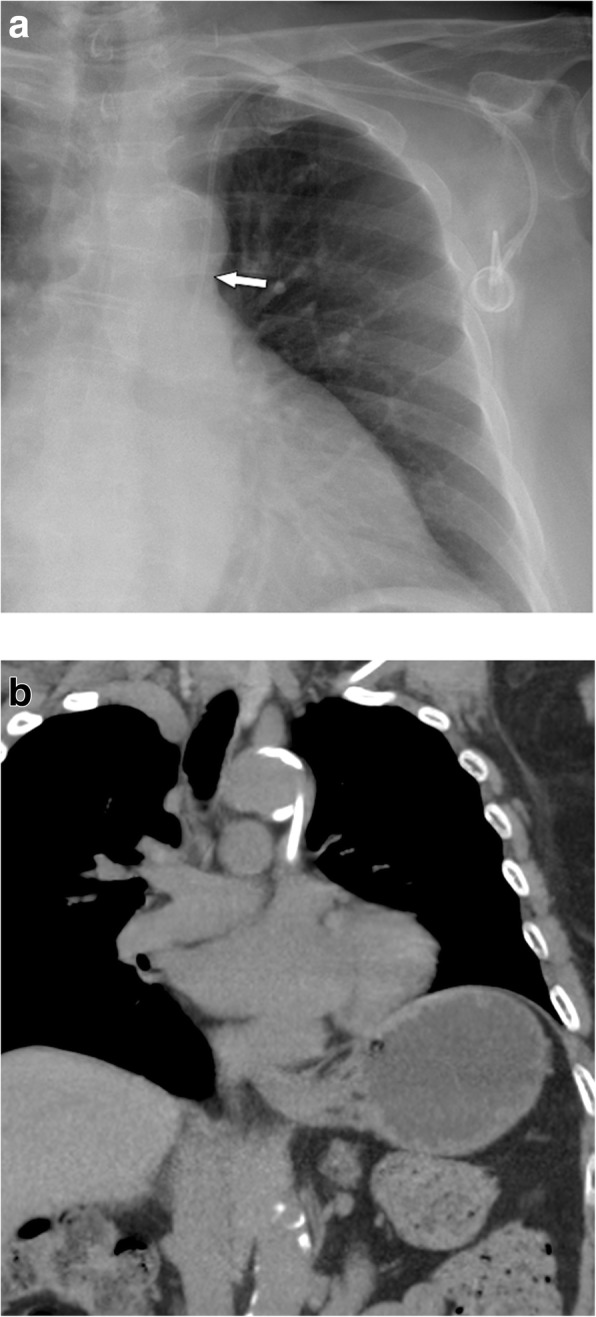
**a** Malpositioning in a persistent left superior vena cava: on a chest film, this could be confused with arterial malpositioning in the aortic arch via the left subclavian artery. **b** Unenhanced CT shows the tip of the catheter in the SVC, which drains into the right atrium

Coursing of the venous tube lateral to the aortic arch could indicate perforation, but may also be seen in patients with persistent left SVC (Fig. 3a, b). Positioning of a port via the left subclavian artery into the left SVC is not malpositioning per se, but venous drainage of the left SVC into the coronary sinus and the right atrium has to be proven. In a few cases, left atrial drainage of persistent left SVC has been shown [17, 18], which may prove disastrous in case of catheter thrombosis or fracture and embolism.

#### Malpositioning into the heart

Periprocedural arrhythmias occur due to placement of guidewire or catheter into the right heart. Atrial arrhythmia is commonly observed during insertion of central venous catheters, with a frequency of up to 41%. Choosing an inappropriate length of the venous tube during insertion may result in positioning of the tip of the catheter in the right atrium, right ventricle, coronary sinus, or even inferior vena cava. Positioning in the right ventricle is associated with an increased risk of damage to the tricuspid valve. Cardiac perforation and tamponade is very rare [19]. Insertion into the coronary sinus may lead to thrombosis. Dislodged catheter fragments can get stuck, making percutaneous retrieval difficult, if not impossible.

**Fig. 4:**
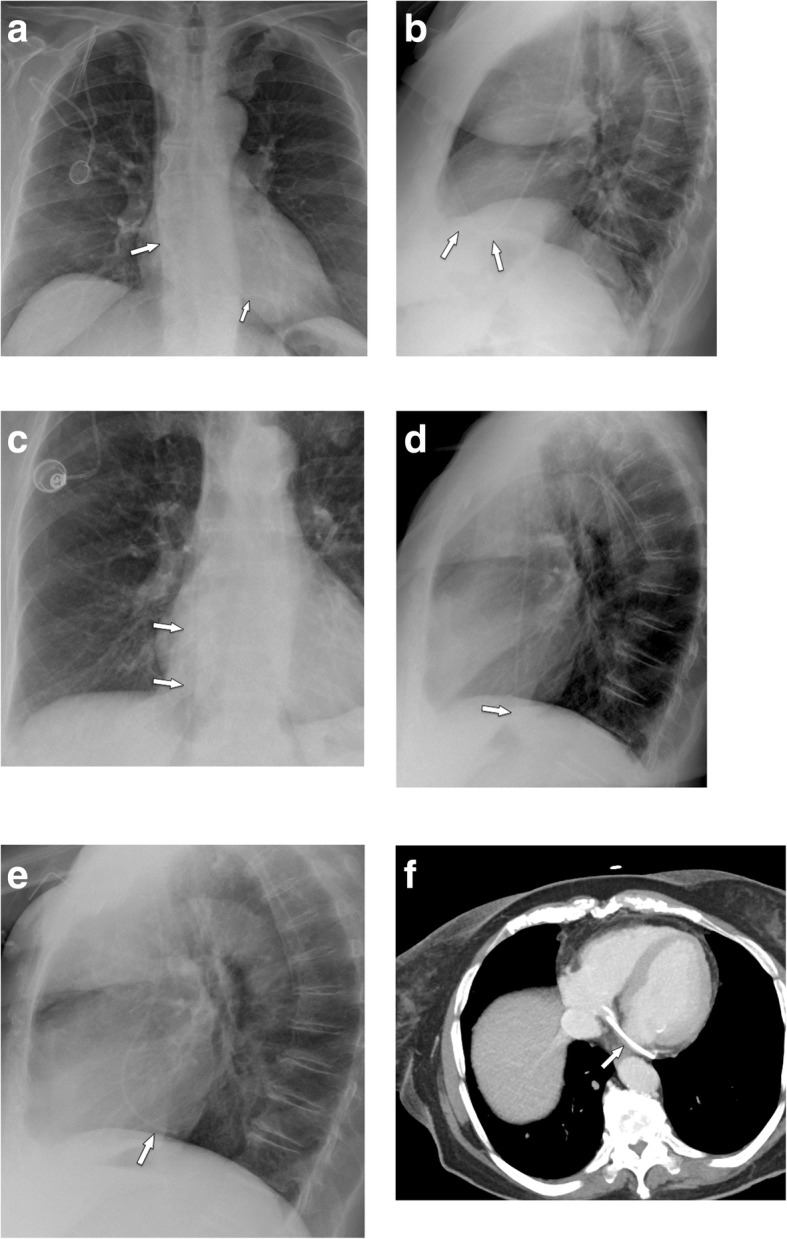
Malpositioning in the heart. **a** PA chest radiography shows a very long, looping catheter, which had not been shortened by the surgeon during the procedure. The tip is projecting onto the right heart (arrow). **b** Lateral chest radiograph shows typical anterior bending (arrows) due to position in the right ventricle. **c** In this example, the catheter tip is not clearly seen, but seems to be placed too deep (arrows). **d** Lateral projection shows a straight course, with the tip in the inferior vena cava (arrow). **e** Five weeks later, a follow-up film now shows posterior curving of the catheter. **f** Contrast-enhanced MDCT (MIP reconstruction) confirms displacement of the catheter into the coronary sinus

Cardiac malpositioning is easily recognized in an AP or PA chest radiograph (Fig. 4a). For assessment of the exact position, the lateral plane is helpful (Fig. 4b–f). It shows a straight course, if the tip is advanced into the IVC, an anterior bend in the right atrium and ventricle, and a sharp posterior curve, if it is placed in the coronary sinus.

#### Arterial and extravascular malpositioning: bleeding and vascular injury

Minor hematomas in the chest wall in the area of port implantation occur in up to 8% and usually regress completely without further treatment [16–18]. Arterial puncture with a small 22-gauge or 25-gauge needle appears in up to 11% and in the vast majority of cases does not cause complications when immediately recognized. However, if incorrect puncture is not noticed, consequent placement of a large-bore dilator or catheter into the artery may result in severe complications with an incidence of 0.1–0.8% [20]. These complications include pseudoaneurysm, arteriovenous fistula, arterial dissection, emboli or thrombosis with stroke, hemothorax with shock, or cervical/mediastinal hematoma, which may lead to airway obstruction [21]. Radiologic recognition of an arterially placed catheter is of utmost importance not only because of the risks during port system use, but also because of the risk of severe hemorrhage during catheter removal.

**Fig. 5:**
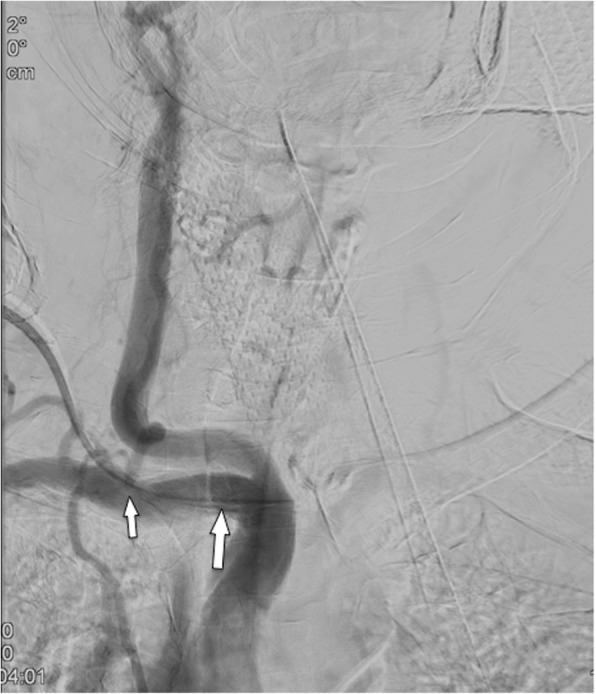
Arterial malpositioning of an interventionally placed port system. DSA shows placement into the subclavian artery close to the origin of the vertebral artery (arrow)

**Fig. 6:**
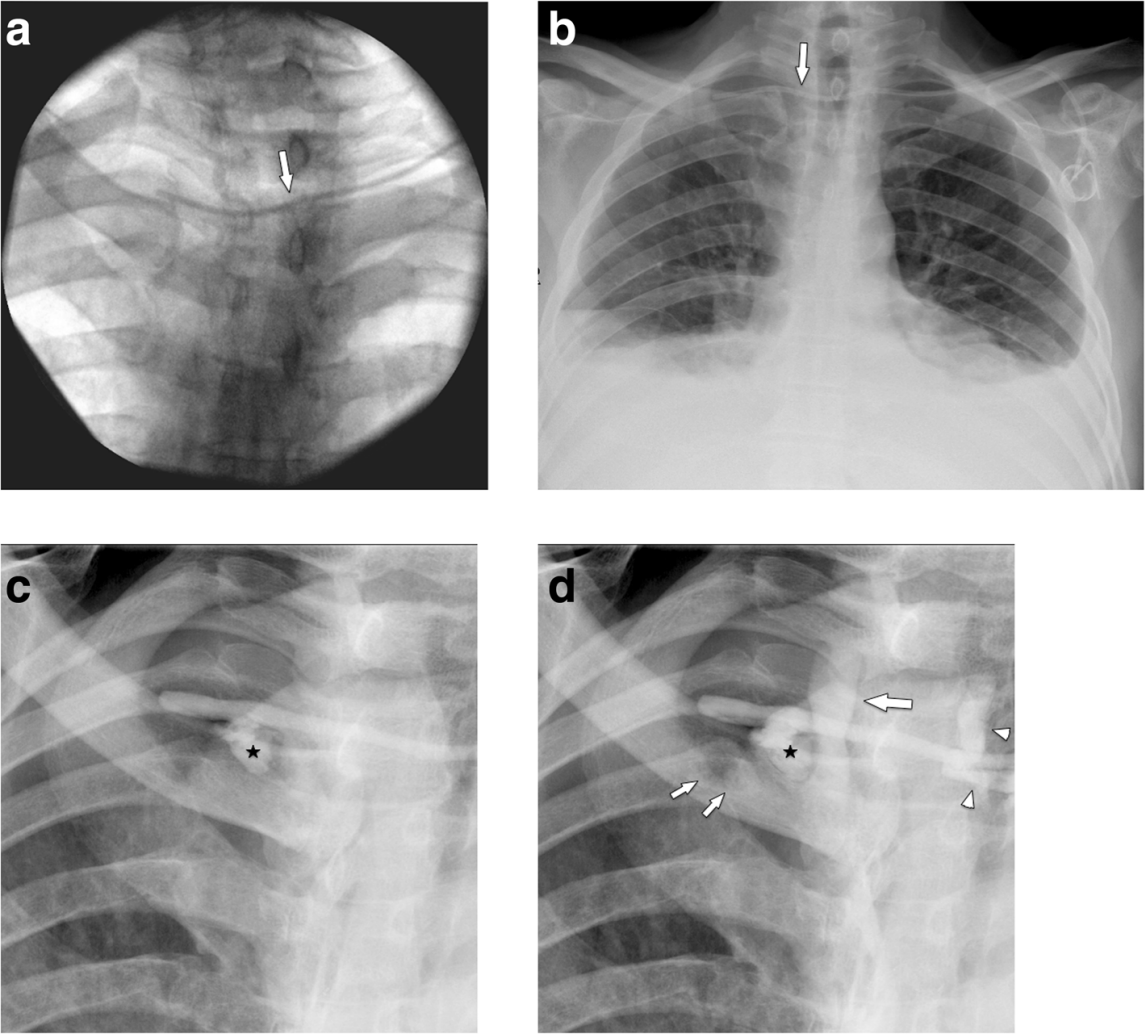
Extravascular malpositioning. **a** Intraoperative fluoroscopy shows the catheter projecting above the clavicles, which is not in line with an intravascular position (in the brachiocephalic vein). **b** Port system was subsequently used for several weeks. Follow-up chest radiograph shows increasing pleural effusions. Malposition of the catheter was reported. **c**, **d** Subsequently a flow confirmation study was performed, which shows extravascular collection of contrast (star) and some contrast material reflux into the internal jugular vein (large arrow), the subclavian vein (small arrow), as well as along the catheter into cervical veins (arrowhead). The catheter has an extravascular course and perforated the contralateral venous angle, with chemotherapy infusions partly entering the venous systems, partly filling up the pleural space

Mediastinal widening or signs of increasing pleural effusions should be considered suspicious for bleeding after implantation (Fig. 5). Common arterial “targets” for arterial malpositioning are the subclavian and the common carotid artery. In such cases, fluoroscopy during the procedure or an AP chest film will show an abnormal course of the catheter, coursing medially toward the aortic arch [22] (Fig. 6a–d). To avoid incorrect puncture/arterial puncture, real-time ultrasound during needle placement should be performed. Furthermore, ultrasound is useful in identifying a normal patent vein before puncture [23, 24]. Several studies report the effectiveness and higher success rate of ultrasound-guided puncture compared to orientation with superficial anatomical landmarks only [25–28].

#### Malpositioning into the thoracic duct

During left subclavian vein catheterization, inadvertent manipulation of the guidewire into the thoracic duct at the venous sinus has been described. Early recognition avoids complications such as chylothorax or infusion into the mediastinum. This potentially serious complication has only been described during central venous catheter placement [29, 30]. To the best of our knowledge, no case of thoracic duct cannulation with a venous port system has been described, although the mechanism would be similar.

#### Pneumothorax

The rate of pneumothorax and hemothorax after puncture of the subclavian vein ranges from 1.5 to 6% and is dependent on the surgeon’s experience. When successfully performing the surgical cut-down of the cephalic vein, there is virtually no risk for pneumothorax or hemothorax. However, latter technique is not possible in some cases and other common complications like dislocation or kinking of the catheter, wound infection, subcutaneous hematoma, or nerve palsy can occur with both techniques [10, 31, 32].

**Fig. 7:**
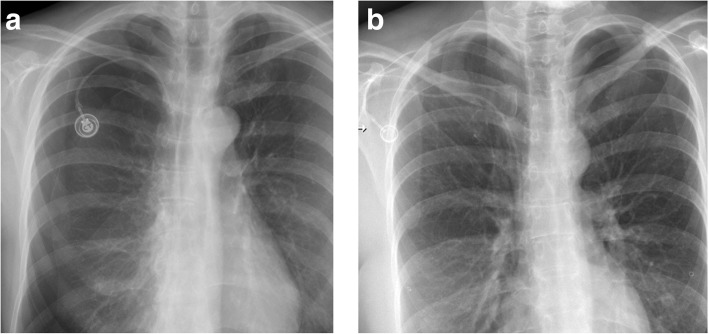
Pneumothorax. **a** Three days after correct placement of port system, the patient complained of dyspnea. Chest radiograph shows a large right pneumothorax. **b** Regular placement of a port system on the right side. There is a contralateral pneumothorax from unsuccessful attempts on the left side, which were not mentioned in the referral

**Fig. 8:**
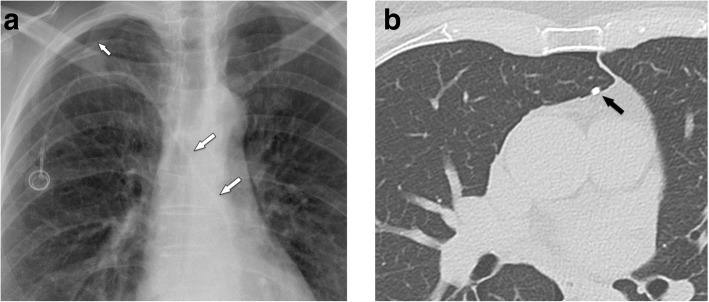
Malpositioning in the pleural space. **a** Postoperative chest radiograph shows an abnormal course of the venous catheter, which crosses the midline. A small apical pneumothorax is noted (arrow). **b** Subsequently, a MDCT was performed, which shows the venous line in the pleural space (arrow) and a pneumothorax

Intraoperative fluoroscopy with its limited image quality does not reliably allow to exclude a pneumothorax. To detect a possible iatrogenic pneumo- or hemothorax, a postoperative chest radiograph is necessary (Figs. 7 and 8). Preferably, chest radiographs should be obtained in upright position and in two projections, because of its higher diagnostic accuracy than supine radiographs.

### Delayed complications

#### Port chamber rotation and thrombosis, catheter pinch-off, fracture, and migration

Mechanical complications include (besides malpositioning in a low-flow vessel) catheter impingement or fragmentation, catheter occlusion, fibrin sheath formation, and damage to the port chamber. These complications lead to system malfunction, which has to be assessed by a flow confirmation study using fluoroscopy or digital subtraction angiography [33]. In a large study [33], in 4.3% of patients, a mechanical complication was suspected for the following reasons: prolonged infusion time, inability to inject saline, subcutaneous extravasation of anticancer drug, arm swelling, neck and/or back pain, and inability to puncture the port.

**Fig. 9:**
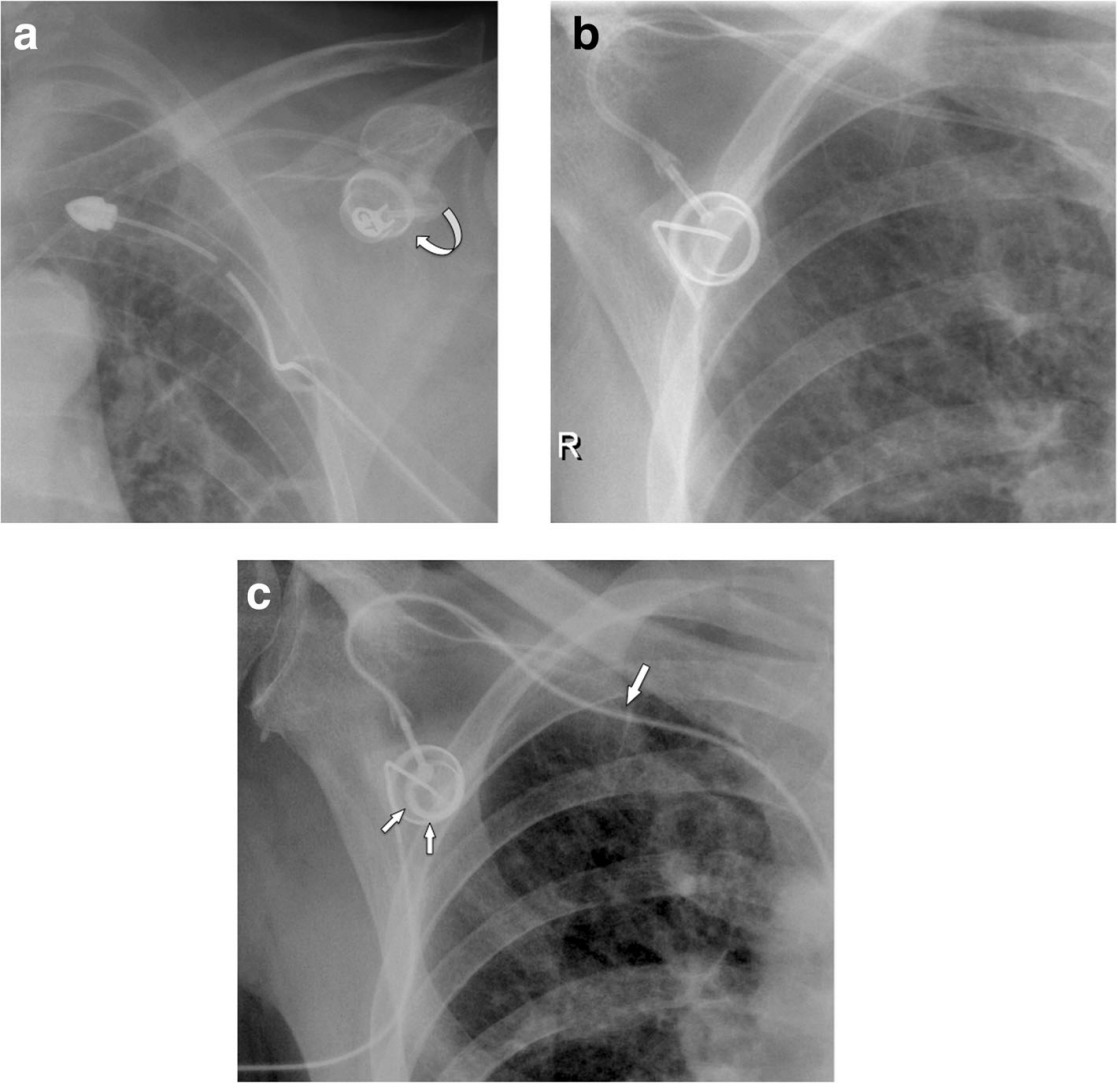
**a** Twisting of port chamber (arrow) results in the inability to puncture the chamber. **b**, **c** Thrombosis of port chamber in another patient with increased flow resistance. Pre-contrast image shows the chamber, which only incompletely and marginally fills with contrast (small arrows). Note that the tube is completely opacified as proof of adequate injection (large error)

**Fig. 10:**
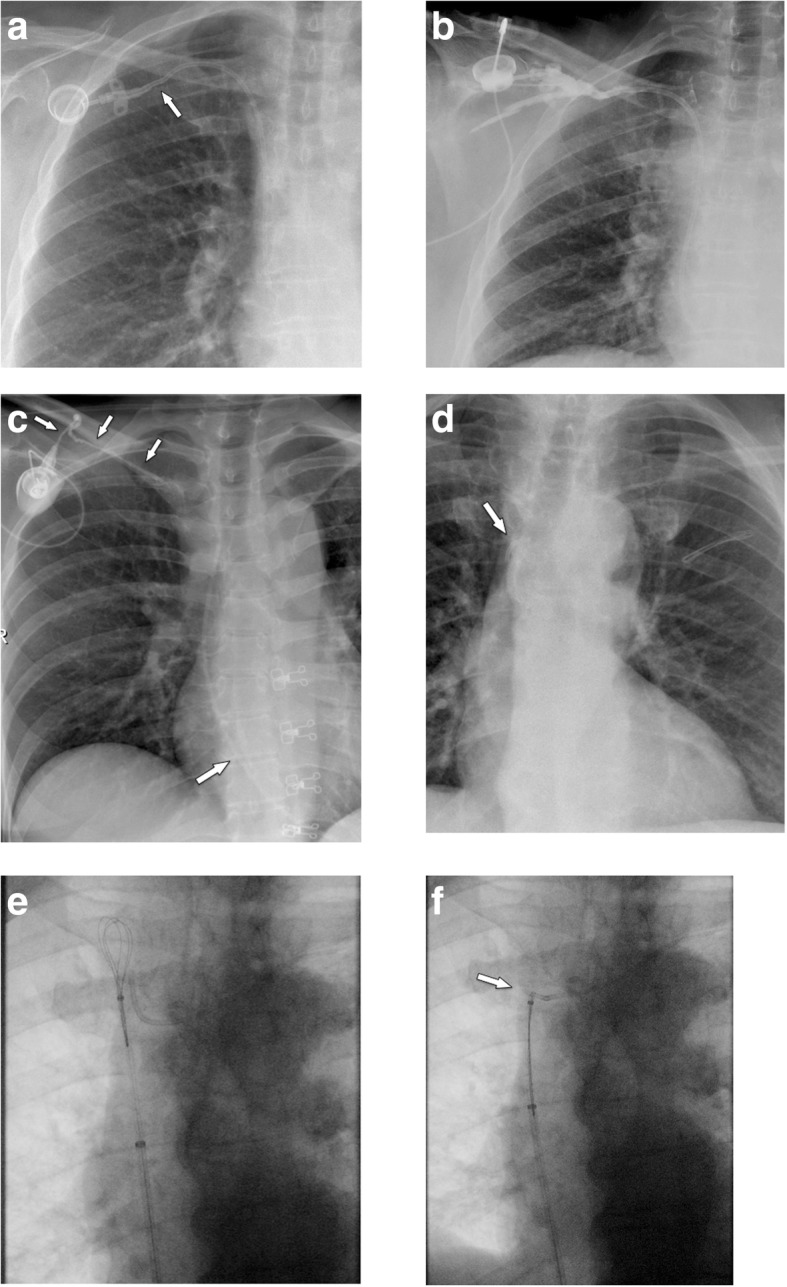
Catheter pinch-off, disconnection, and fracture. **a**, **b** Catheter pinch-off with subsequent fracture. **a** Chest radiographs show a slight kink and pinch-off of the catheter at the thoracic inlet (between clavicle and first rib). **b** Five months later, a flow confirmation study shows extravasation at the thoracic inlet (due to fracture). Incidental note is made of the catheter tip in the right atrium. **c** Catheter disconnection in another patient: the entire catheter has gone off and embolized in the heart (large arrow). Only the subcutaneous fibrous tunnel is opacified with contrast (small arrows). **d**–**f** Fracture of catheter and percutaneous removal. **d** After explantation of a port system, a catheter fragment is still visible (arrow). **e** Via a right femoral vein access a snare is advanced toward the catheter tip. **f** After pulling the snare tight around the catheter (arrow), it can be safely removed

If the port chamber cannot be punctured, careful inspection during fluoroscopy is sought. The chamber may have twisted (Fig. 9a), especially if it has not been sutured to the fascia (which is the case in radiologic interventional implantation). This may occur early or later after implantation. Inability to aspirate blood or increased resistance during infusion is often related to thrombosis of the port chamber (Fig. 9b, c), catheter pinch-off at the thoracic inlet (Fig. 10a), catheter disconnection (Fig. 10b), or catheter fragmentation (Fig. 10c). Catheter disconnection and fragmentation may lead to embolism of fragments into the right heart or even the pulmonary artery, with potentially devastating consequences, such as life-threatening tachycardia, cardiac perforation, or pulmonary pseudoaneurysms [34, 35]. In case of catheter disconnection or fragmentation with embolization, percutaneous retrieval via the femoral vein is the method of choice [36]. With a guiding catheter, a gooseneck snare is maneuvered toward the fragment to catch the tip. Once the snare is pulled tight around the catheter (fragment), it can safely be retrieved (Fig. 10d–f). In patients with delayed diagnosis of catheter fragmentation, fibrin sheath formation around the catheter with adhesion to the vessel or endocardium may prevent extraction [36].

#### Venous thrombosis

**Fig. 11:**
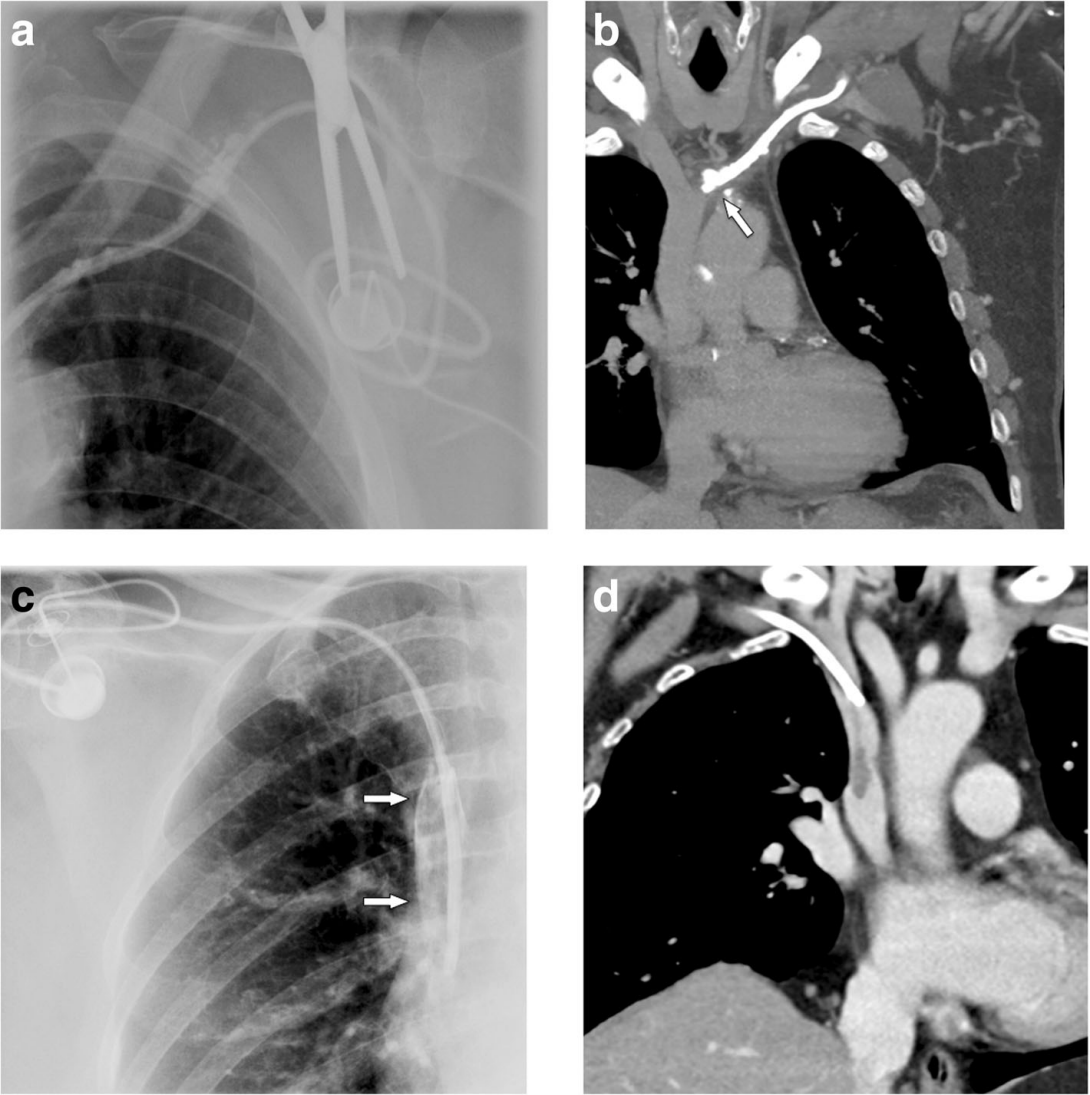
Thrombosis. **a** Flow confirmation study shows contrast backflow around the catheter. It is not clear, whether the catheter is in an intravascular position. **b** CT was subsequently performed, which shows the tip of the catheter too high in the brachiocephalic vein with venous thrombosis and development of multiple collaterals. **c** In another patient with increased resistance during infusions, flow confirmation study shows a fibrin sheath around the catheter (arrows). Infusion of thrombolytics (rTPA) restored good flow of the system. **d** In another patient, incidental finding of a thrombus at the catheter tip detected during contrast-enhanced MDCT follow-up

In a large series [37] on 51,049 patients, 1.81% of patients developed an upper extremity thrombosis. Risk factors included age < 65, presence of more comorbidities, history of any deep venous thrombosis, non-white race, and presence of certain malignancies (such as lung cancer and gastrointestinal cancer). Thrombotic complications of port systems occur in two forms: stenosis or occlusion of the host vein due to trauma to the venous wall or thrombus formation around the catheter tip [12]. The former may be caused by manipulation at the vascular entry site. Another important risk factor is malpositioning of the catheter tip into a smaller caliber, low-flow vein, such as the brachiocephalic or subclavian vein (“catheter too short”) (Fig. 11a, b). The latter form is caused by a pro-coagulative state, which leads to formation of a “fibrin sheath” around the catheter (Fig. 11c, d). This may result in increased flow resistance during infusions. Short infusions of thrombolytics restore tube patency with a high success rate [38]. However, such a fibrin sheath is also the breeding ground for microorganisms and subsequent biofilm formation and infections [12].

#### Infection

Infections are the most common complication after implantation of a venous port system [39, 40]. Infections of port venous systems include pocket and/or tunnel cellulitis or the more common catheter-related blood stream infections. Latter are diagnosed after exclusion of other sources of infection or via blood culture. Incidence of port-associated infection ranges from 0.6 to 27% [9]. In the study of Shim et al. [41], 45 out of 1747 implanted port systems were explanted due to infection. The most common causative microorganisms were *Staphylococcus* species, *Candida* species, and non-tuberculosis *Mycobacterium*. In patients with good general condition, intravenous broad-spectrum antibiotic therapy may be attempted until specific microorganisms are identified and therapy can be adapted. In the vast majority of cases, antibiotic therapy may save the port system. More complex and difficult to handle are relapsing infections in immunocompromised patients, infections with fungal species, or septic complications such as endocarditis or local abscess formations [42–44]. Other factors that may influence rate of infections include heavy microbial colonization of insertion site, neutropenia, and duration of device usage: portal venous systems carry a bloodstream infection rate of 2.81 cases per 1000 days. In general, implantable devices have a lower rate of infection than non-tunneled central lines [45, 46]. As described, thrombus or fibrin sheath formation can provide a biofilm for microorganisms. Hence, there is a causal relationship between catheter-related thrombosis and catheter-related infections.

#### Air embolism

Venous air embolism can occur during implantation, explantation, and use of central venous catheters. Clinical appearance ranges from asymptomatic to cardiovascular collapse and death. The development and severity of possible cardiovascular and pulmonary symptoms depend upon volume of air aspirated into the venous system. The lethal dose to humans is theorized to be 3–5 ml/kg b.w. Clinical history is the most important factor for diagnosing embolism, because the suspicion of venous gas embolism is based on the temporal relationship between the invasive procedure and appearance of clinical symptoms. A useful maneuver to avoid air embolism is placing the patient in Trendelenburg position [22, 47].

#### Postoperative complications: is a routine chest radiograph necessary?

The reported incidence of complications occurring in central venous port systems varies widely, ranging from 1.28 [48] to 7.2% [15] in large series, with infections being the most common. In a retrospective study conducted in the USA, the immediate postoperative complication rate was 0.58%. This led the authors to conclude that the routine use of postoperative chest radiographs could be discontinued because of high costs (average cost, US $345—per patient) and low benefit [49]. However, in Europe, the cost for a chest radiograph is considerably lower than reported in that study and the clinical benefit is in clinical practice probably much higher. The very low incidence of complications reported (0.58%) is due to the study design: only procedural abnormalities noted intraoperatively were recorded. All malpositioning not detected immediately by the operator was not included in the analysis. However, even severe malpositioning may escape intraoperative recognition (Fig. 6). In another study, a very low incidence of procedural complications was detected on chest radiographs (0.34%) based on interpretation by the surgeon, with only two cases of malpositioning reported in 891 patients [50]. Thus, given the clinical importance of proper placement of a central port system, post-procedural radiographic documentation either in the angiography suite or by chest films (after surgical placement) and reporting of the study by a radiologist seems to be indispensable.

In conclusion, central venous port systems have gained a significant role in the treatment of many patients, who require long-term intravenous therapy. Radiologic assessment is of key importance to detect complications, such as malpositioning, vein perforation and bleeding, pneumothorax, and thrombosis. Imaging is also important to plan interventional procedures for retrieval of fractured and embolized catheter fragments.

## Data Availability

Not applicable

## References

[1] Tabatabaie Omidreza, Kasumova Gyulnara G., Eskander Mariam F., Critchlow Jonathan F., Tawa Nicholas E., Tseng Jennifer F. (2017). Totally Implantable Venous Access Devices. American Journal of Clinical Oncology.

[2] Nakamura Takatoshi, Sasaki Jiichiro, Asari Yasushi, Sato Takeo, Torii Shinzo, Watanabe Masahiko (2017). Complications after implantation of subcutaneous central venous ports (PowerPort Ⓡ ). Annals of Medicine and Surgery.

[3] Hamilton HC, Foxcroft DR (2007) Central venous access sites for the prevention of venous thrombosis, stenosis and infection in patients requiring long-term intravenous therapy. Cochrane Database Syst Rev 3:Cd004084 10.1002/14651858.CD004084.pub2 17636746

[4] Simpson KR, Hovsepian DM, Picus D (1997). Interventional radiologic placement of chest wall ports: results and complications in 161 consecutive placements. J Vasc Interv Radiol.

[5] Ballarini C, Intra M, Pisani Ceretti A (1999). Complications of subcutaneous infusion port in the general oncology population. Oncology.

[6] Denys BG, Uretsky BF, Reddy PS (1993). Ultrasound-assisted cannulation of the internal jugular vein. A prospective comparison to the external landmark-guided technique. Circulation.

[7] Kim DH, Ryu DY, Jung HJ, Lee SS (2019). Evaluation of complications of totally implantable central venous port system insertion. Exp Ther Med.

[8] Skelton WP 4th, Franke AJ, Welniak S et al (2019) Investigation of complications following port insertion in a cancer patient population: a retrospective analysis. Clin Med Insights Oncol 24:13 10.1177/1179554919844770 PMC6482646 31040735

[9] Yildizeli B., Laçin T., Batirel H.F., Yüksel M. (2004). Complications and management of long-term central venous access catheters and ports. The Journal of Vascular Access.

[10] Balestreri L, De Cicco M, Matovic M, Coran F, Morassut S (1998) Totally implantable central venous access ports for long-term chemotherapy. A prospective study analyzing complications and costs of 333 devices with a minimum follow-up of 180 days. Ann Oncol 9:767–773 10.1023/a:1008392423469 9739444

[11] Balestreri Luca, De Cicco Marcello, Matovic Mira, Coran Francesco, Morassut Sandro (1995). Central venous catheter-related thrombosis in clinically asymptomatic oncologic patients: a phlebographic study. European Journal of Radiology.

[12] Walser EM (2012). Venous access ports: indications, implantation technique, follow-up, and complications. Cardiovasc Intervent Radiol.

[13] Keulers AR, Kiesow L, Mahnken AH (2018). Port implantation in patients with severe thrombocytopenia is safe with interventional radiology. Cardiovasc Intervent Radiol.

[14] Teichgräber UK, Pfitzmann R, Hofmann HA (2011) Central venous port systems as an integral part of chemotherapy. Dtsch Arztebl Int 108:147–153 quiz 154 10.3238/arztebl.2011.0147 PMC3063378 21442071

[15] Kakkos A, Bresson L, Hudry D (2017). Complication-related removal of totally implantable venous access port systems: does the interval between placement and first use and the neutropenia-inducing potential of chemotherapy regimens influence their incidence? A four-year prospective study of 4045 patients. Eur J Surg Oncol.

[16] Wall C, Moore J, Thachil J (2016). Catheter-related thrombosis: a practical approach. J Intensive Care Soc.

[17] Yousaf M, Malak SF (2008). Left atrial drainage of a persistent left superior vena cava. Radiol Case Rep.

[18] Metzler B, Hillebrand H, Eulenbruch H.-P, Dierkesmann R, Hust M H (2002). Persistierende linke Vena cava superior mit Rechts-Links-Shunt zum linken Atrium. DMW - Deutsche Medizinische Wochenschrift.

[19] Shields LB, Hunsaker DM, Hunsaker JC (2003). Iatrogenic catheter-related cardiac tamponade: a case report of fatal hydropericardium following subcutaneous implantation of a chemotherapeutic injection port. J Forensic Sci.

[20] Guilbert MC, Elkouri S, Bracco D (2008). Arterial trauma during central venous catheter insertion: case series, review and proposed algorithm. J Vasc Surg.

[21] Keum DY, Kim JB, Chae MC (2013). Safety of a totally implantable central venous port system with percutaneous subclavian vein access. Korean J Thorac Cardiovasc Surg.

[22] Zoltowska D, Kalavakunta J (2018). A port-a-cath in aorta. Clin Case Rep.

[23] Lalu MM, Fayad A, Ahmed O (2015). Ultrasound-guided subclavian vein catheterization: a systematic review and meta-analysis. Crit Care Med.

[24] Byon HJ, Lee GW, Lee JH (2013). Comparison between ultrasound-guided supraclavicular and infraclavicular approaches for subclavian venous catheterization in children—a randomized trial. Br J Anaesth.

[25] Mussa Firas F., Towfigh Shirin, Rowe Vincent L., Major Kevin, Hood Douglas B., Weaver Fred A. (2006). Current Trends in the Management of Iatrogenic Cervical Carotid Artery Injuries. Vascular and Endovascular Surgery.

[26] Denys BG, Uretsky BF, Reddy PS, Ruffner RJ, Sandhu JS, Breishlatt WM (1991) An ultrasound method for safe and rapid central venous access. N Engl J Med 324:566 10.1056/NEJM199102213240816 1992315

[27] Gilbert TB, Seneff MG, Becker RB (1995). Facilitation of internal jugular venous cannulation using an audio-guided Doppler ultrasound vascular access device: results from a prospective, dual-center, randomized, crossover clinical study. Crit Care Med.

[28] Hayashi H, Amano M (2002). Does ultrasound imaging before puncture facilitate internal jugular vein cannulation? Prospective randomized comparison with landmark-guided puncture in ventilated patients. J Cardiothorac Vasc Anesth.

[29] Teichgraber UK, Nibbe L, Gebauer B, Wagner HJ (2003) Inadvertent puncture of the thoracic duct during attempted central venous catheter placement. Cardiovasc Intervent Radiol 26:569–571 10.1007/s00270-003-0102-1 15061186

[30] Májek M., Malatinsky J., Kadlic T. (1977). Inadvertent Thoracic Duct Catheterization during Transjugular Central Venous Cannulation. A Case Report. Acta Anaesthesiologica Scandinavica.

[31] Biffi R., Pozzi S., Pace U., Cenciarelli S., Zambelli M., Andreoni B. (2001). Treatment of Pneumothorax as a Complication of Long-Term Central Venous Port Placement in Oncology Patients. An Observational Study. The Journal of Vascular Access.

[32] McGee DC, Gould MK (2003). Preventing complications of central venous catheterization. N Engl J Med.

[33] Sofue Keitaro, Arai Yasuaki, Takeuchi Yoshito, Sugimura Kazuro (2013). Flow confirmation study for central venous port in oncologic outpatient undergoing chemotherapy: Evaluation of suspected system-related mechanical complications. European Journal of Radiology.

[34] Oz Kursad, Demirhan Recep, Onan Burak, Sancakli Irfan (2009). Pulmonary Artery Pseudoaneurysm After a Vascular Access Port Catheter Implantation. The Annals of Thoracic Surgery.

[35] Gowda MR, Gowda RM, Khan IA (2004). Positional ventricular tachycardia from a fractured mediport catheter with right ventricular migration—a case report. Angiology.

[36] Gebauer Bernhard, Teichgräber Ulf Karl, Podrabsky Petr, Werk Michael, Hänninen Enrique Lopez, Felix Roland (2007). Radiological Interventions for Correction of Central Venous Port Catheter Migrations. CardioVascular and Interventional Radiology.

[37] Tabatabaie O, Kasumova GG, Kent TS (2017). Upper extremity deep venous thrombosis after port insertion: what are the risk factors?. Surgery.

[38] Daeihagh Pirouz, Jordan Jennifer, Chen G.John, Rocco Michael (2000). Efficacy of tissue plasminogen activator administration on patency of hemodialysis access catheters. American Journal of Kidney Diseases.

[39] Teichgräber Ulf K. M., Kausche Stephan, Nagel Sebastian N., Gebauer Bernhard (2011). Outcome analysis in 3,160 implantations of radiologically guided placements of totally implantable central venous port systems. European Radiology.

[40] Chang Lilu, Tsai Jir-Shiong, Huang Shin-Ju, Shih Chiang-Ching (2003). Evaluation of infectious complications of the implantable venous access system in a general oncologic population. American Journal of Infection Control.

[41] Shim J, Seo T, Song M (2014). Incidence and risk factors of infectious complications related to implantable venous-access ports. Korean J Radiol.

[42] Bouza E., Burillo A., Muñoz P. (2002). Catheter-related infections: diagnosis and intravascular treatment. Clinical Microbiology and Infection.

[43] O'Grady NP, Alexander M, Burns LA (2011). Guidelines for the prevention of intravascular catheter-related infections. Clin Infect Dis.

[44] Fätkenheuer G, Buchheidt D, Cornely OA et al (2003) Central venous catheter (CVC)-related infections in neutropenic patients—guidelines of the infectious diseases working party (AGIHO) of the german society of hematology and oncology (DGHO). Ann Hematol 82(Suppl 2):S149–S157 10.1007/s00277-003-0769-z 13680168

[45] Yoshida Junichi, Ishimaru Toshiyuki, Kikuchi Tetsuya, Matsubara Nobuo, Asano Ikuyo (2011). Association between risk of bloodstream infection and duration of use of totally implantable access ports and central lines: A 24-month study. American Journal of Infection Control.

[46] Leonidou L, Gogos CA (2010). Catheter-related bloodstream infections: catheter management according to pathogen. Int J Antimicrob Agents.

[47] Gordy S, Rowell S (2013). Vascular air embolism. Int J Crit Illn Inj Sci.

[48] Tsutsumi S, Fukasawa T, Fujii T (2012). Central venous port system-related complications in outpatient chemotherapy for colorectal cancer. Hepatogastroenterology.

[49] Vaughan JG, Cauthen AB, Allen A, Dale P (2017) Evaluation of cardiopulmonary complication rates after port insertion: is a postoperative X-ray needed? Am Surg 83:778–779 28738951

[50] Thomopoulos T, Meyer J, Staszewicz W (2014). Routine chest X-ray is not mandatory after fluoroscopy-guided totally implantable venous access device insertion. Ann Vasc Surg.

